# Phage biocontrol of enteropathogenic and shiga toxin-producing *Escherichia coli* in meat products

**DOI:** 10.3389/fcimb.2013.00020

**Published:** 2013-06-06

**Authors:** David Tomat, Leonel Migliore, Virginia Aquili, Andrea Quiberoni, Claudia Balagué

**Affiliations:** ^1^Área de Bacteriología, Facultad de Ciencias Bioquímicas y Farmacéuticas, Universidad Nacional de RosarioRosario, Argentina; ^2^Facultad de Ingeniería Química, Instituto de Lactología Industrial (UNL - CONICET)Santa Fe, Argentina

**Keywords:** *Escherichia coli*, bacteriophage, phage biocontrol, bacteriophage insensitive mutant, phage cocktail

## Abstract

Ten bacteriophages were isolated from faeces and their lytic effects assayed on 103 pathogenic and non-pathogenic *Enterobacteriaceae*. Two phages (DT1 and DT6) were selected based on their host ranges, and their lytic effects on pathogenic *E*. *coli* strains inoculated on pieces of beef were determined. We evaluated the reductions of viable cells of *Escherichia coli* O157:H7 and non-O157 Shiga toxigenic *E. coli* strains on meat after exposure to DT6 at 5 and 24°C for 3, 6, and 24 h and the effect of both phages against an enteropathogenic *E. coli* strain. Significant viable cell reductions, compared to controls without phages, at both temperatures were observed, with the greatest decrease taking place within the first hours of the assays. Reductions were also influenced by phage concentration, being the highest concentrations, 1.7 × 10^10^ plaque forming units per milliliter (PFU/mL) for DT1 and 1.4 × 10^10^ PFU/mL for DT6, the most effective. When enteropathogenic *E. coli* and Shiga toxigenic *E. coli* (O157:H7) strains were tested, we obtained viable cell reductions of 0.67 log (*p* = 0.01) and 0.77 log (*p* = 0.01) after 3 h incubation and 0.80 log (*p* = 0.01) and 1.15 log (*p* = 0.001) after 6 h. In contrast, all nonpathogenic *E. coli* strains as well as other enterobacteria tested were resistant. In addition, phage cocktail was evaluated on two strains and further reductions were observed. However, *E. coli* bacteriophage insensitive mutants (BIMs) emerged in meat assays. BIMs isolated from meat along with those isolated by using the secondary culture method were tested to evaluate resistance phenotype stability and reversion. They presented low emergence frequencies (6.5 × 10^−7^–1.8 × 10^−6^) and variable stability and reversion. Results indicate that isolated phages were stable on storage, negative for all the virulence factors assayed, presented lytic activity for different *E. coli* virotypes and could be useful in reducing Shiga toxigenic *E. coli* and enteropathogenic *E. coli* viable cells in meat products.

## Introduction

Shiga toxin-producing *Escherichia coli* (STEC) are human pathogens that can cause diarrhea, as well as severe clinical manifestations including hemorrhagic enterocolitis, hemolytic uremic syndrome (HUS), and thrombotic thrombocytopenic purpura (Su and Brandt, 1995; Griffin et al., 2002; Yoon and Hovde, 2008). STEC produce several virulence factors which contribute to their pathogenicity. Shiga toxins (Stx), AB type toxins that inhibit protein synthesis in target cells, are the most characterized virulence factors (Thorpe et al., 2002). Shiga toxins produced in the intestines by STEC are able to enter the systemic circulation causing severe damage to distal organs. The degree of damage is related to the amount of toxin produced during the infection (Ritchie et al., 2003). STEC synthesize two main types of Shiga toxins encoded by *stx1* and *stx2* genes. Moreover, the enterocyte attaching-and-effacing lesion gene (*eaeA*), which is also present in enteropathogenic strains (EPEC), can contribute to the virulence of STEC. The gene codes for the intimin protein, which allows bacteria to attach themselves to the intestinal epithelium (Frankel et al., 1998).

Foodborne disease-producing *Enterobacteriaceae*, such as *Shigella* spp., *Salmonella* spp., EPEC and STEC, are important etiologic agents of infantile gastroenteritis in Argentina (Binsztein et al., 1999; Rivas et al., 2008). In developing countries, EPEC are the cause of outbreaks of infantile diarrhea with high mortality in children under two years of age. In Argentina, HUS is endemic, with approximately 400 new cases being reported annually by National Health Surveillance System (Rivas et al., 2006), and more than 7000 cases being reported since 1965 (NCASP, 1995). In 2005, the annual incidence of HUS is 13.9 cases/100,000 children under five years of age (Rivas et al., 2006). Recent epidemiological studies showed that there is a sustained global increase in the isolation of non-O157 STEC strains from humans (Tozzi et al., 2003; Brooks et al., 2005; Bettelheim, 2007) and animals (Jenkins et al., 2003; Fernandez et al., 2009), particularly STEC of serogroups O26, O103, and O111 (Ogura et al., 2007).

The therapeutic potential of bacteriophages has been explored since they were discovered by Felix d'Herelle (Summers, 1999). Some of the attributes that make bacteriophages interesting as tools for biological control are: (i) their ability to infect and lyse specific bacterial target cells and their inability to infect eukaryotic cells; (ii) phages generally do not cross bacterial species or genus barriers, and therefore do not affect desirable microorganisms commonly present in foods, the gastrointestinal tract or the normal bacterial microbiota (Carlton et al., 2005); (iii) phages need a bacterial host in which to multiply and therefore will persist only as long as the sensitive host is present (Clark and March, 2006). The potential of bacteriophages to control food pathogens is reflected in recent studies involving various pathogens including *Campylobacter jejuni* (Atterbury et al., 2003; Bigwood et al., 2008), *E. coli* O157:H7 (O'Flynn et al., 2004; Abuladze et al., 2008) and *Listeria monocytogenes* (Leverentz et al., 2003; Guenther et al., 2009; Holck and Berg, 2009). Several strategies are currently being applied to preserve perishable refrigerated foods and extend their shelf-life. However, physical processes and chemical compounds (preservatives) used for this purpose may alter meat organoleptic properties. Although bacteriophages represent a novel approach, there are no reports of their industrial use to improve safety, even if this “new, ecological, and specific” technology may be cheaper than “older” technologies, since phages can be isolated from the environment and are self-replicating entities. On the other hand, their inclusion into a meat product can be seen as a less aggressive approach.

The aim of this work was to isolate phages with specific lytic capacity for *E. coli* strains in order to determine phage host range and analyze their potential as biocontrol agents for STEC and EPEC strains in beef products.

## Materials and methods

### Bacteriophage isolation and preparation of stocks

*E. coli* DH5α was used to isolate bacteriophages from fifty stool samples of patients with diarrhea treated at the Centenary Hospital, Rosario. This strain was grown up to an optical absorbance of 1 (A_600_ = 1) in 10 mL of Hershey broth (8 g/L Bacto nutrient broth, 5 g/L Bacto peptone, 5 g/L NaCl, and 1 g/L glucose) (Difco, Detroit, MI, USA) supplemented with MgSO_4_ (5 mM) (Cicarelli, San Lorenzo, Santa Fe, Argentina). A portion of faeces (5 g) was added and the culture was incubated for a further 12 h at 37°C. Next, chloroform (0.5 mL, Cicarelli) was added and the preparation was mixed and centrifuged at 15,000× g for 10 min. The supernatant was then filtered through a 0.45 μm pore size (Gamafil S.A., Buenos Aires, Argentina) (Kudva et al., 1999). Bacteriophage isolation and purification were performed by the double-layer plaque technique (Balagué et al., 2006). Briefly, aliquots of filtrates (10 and 100 μL) were mixed with 100 μL of recipient strain culture (A_600_ = 1), three mL of molten soft agar at 45°C (Hershey broth supplemented with 5 mM MgSO_4_ and 0.7% agar) were added to each suspension and the mixture was poured onto pre-solidified Hershey agar plates and incubated overnight at 37°C. To isolate and purify phages, well-defined single plaques on the soft agar were picked and placed in 5 mL of Hershey medium supplemented with 5 mM MgSO_4_. Tubes were kept at 4°C for 2 h and then inoculated with 100 μL of recipient strain culture (A_600_ = 1). Inoculated tubes were incubated at 37°C with intermittent shaking until complete lysis. Next, chloroform (0.1 mL) was added and cultures were centrifuged at 4000× g for 10 min. Phage stocks were stored at 4°C and enumerated by the double-layer plaque technique (Jamalludeen et al., 2007). These steps were repeated three times. Stability of phage stocks was evaluated after two months of storage at 4°C.

### Bacteriophage and bacteria characterization

Phage electron micrographs were obtained by the procedure of Bolondi et al. (1995). Phage suspensions were concentrated by centrifugation (1 h, 70,000 × g, 5°C) and subsequently stained with phosphotungstic acid (2% w/v) (Biopack, Buenos Aires, Argentina). Electron micrographs were obtained using a JEOL 1200 EX II electron microscope (INTA Castelar, Buenos Aires, Argentina) operating at 85 kV. Phage morphologies and dimensions (head diameter, tail length, and diameter) were recorded.

Phages and strains of *E. coli* were tested for the presence of toxin-encoding genes (*stx1*, Shiga toxin 1; *stx2*, Shiga toxin 2; *eaeA*, attaching-and effacing; *LT1*, thermolabile toxin and *ST1*, thermostable toxin) of diarrheogenic *E. coli* by the polymerase chain reaction (PCR) using primers detailed in Table 1 (Pass et al., 2000). PCR conditions were as follows: initial denaturing step at 95°C for 2 min, followed by 25 cycles of 95°C for 30 s, annealing at 63°C for 30 s and elongation at 72°C for 30 s, followed by a final step at 72°C for 5 min to achieve complete product elongation. *E. coli* ATCC43889 (*stx2* and *eaeA*), ATCC43890 (*stx1*), and ATCC43895 (*stx1*, *stx2*, and *eaeA*, and also harboring the *stx2* phage, 933W) were used as positive controls, while enterotoxigenic *E. coli* ATCC35401 was used for *LT1* and *ST1* genes. *E. coli* HB101 and ATCC98222 were utilized as negative controls. Amplified products were resolved by electrophoresis using 3% agarose gels in TBE buffer (89 mM Tris borate, 2 mM EDTA, pH 8.0) (Promega, Madison, WI, USA) at 100 V for 3 h. Gels were stained with ethidium bromide (0.5 μg/mL) (Sigma, St. Louis, MO, USA) and PCR products were visualized under UV light.

**Table 1:** **Sequences of primers used in this study**.

**Gene**	**Primer^a^**	**Product size**
		**(bp) expected**
*stx1*	fp: 5′-ACGTTACAGCGTGTTGCRGGGATC-3′	121
	bp: 5′-TTGCCACAGACTGCGTCAGTRAGG-3′	
*stx2*	fp: 5′-TGTGGCTGGGTTCGTTTATACGGC-3′	102
	bp: 5′-TCCGTTGTCATGGAAACCGTTGTC-3′	
*eaeA*	fp: 5′-TGAGCGGCTGGCATGATGCATAC-3′	241
	bp: 5′-TCGATCCCCATCGTCACCAGAGG-3′	
*LT1*	fp: 5′-TGGATTCATCATGCACCACAAGG-3′	360
	bp: 5′-CCATTTCTCTTTTGCCTGCCATC-3′	
*ST1*	fp: 5′-TTTCCCCTCTTTTAGTCAGTCAACTG-3′	160
	bp: 5′-GGCAGGACTACAACAAAGTTCACAG-3′	

a *fp, forward primer; bp, backward primer. stxl and stx2: Shiga toxin1 and 2 encoding genes; eaeA: intimin encoding gene; LTl and STl: thermolabile and thermostable toxins encoding genes*.

### Bacteriophage specificity

The host range of each phage was determined by the double layer agar technique using 44 strains isolated from stool samples, and urine cultures (uropathogenic *E. coli*, UPEC). Stool and urine samples were streaked in Cystine Lactose Electrolyte Deficient (CLED) agar plates. Simmons citrate agar test was performed on growing lactose positive colonies. After incubation for 24 h at 35°C, only lactose positive and citrate negative colonies were further identified using API system (Biomerieux, Bs. As., Argentina). Sixteen *E. coli* strain from food (Balagué et al., 2006), one uropathogenic *E*. *coli* strain (*E. coli* T149) which expresses fimbriae P and α-hemolysin (Balagué et al., 2004) and five ATCC *E. coli* strains were also tested (ATCC 43890; 43889; 43895; 35401 and 98222). Previously characterized (API system) isolates from stool samples were also tested: *Shigella flexneri*, *S. sonnei*, *Proteus mirabilis*, *Citrobacter freundii*, *Klebsiella pneumoniae*, *Salmonella enteritidis*, *Salmonella* Typhi and *Salmonella* Typhimurium. Strains tested against stock phages are listed in Table 2. Bacteriophage sensitivity was assayed by placing 10 μL of phage suspension on the solidified soft-agar layer inoculated with 100 μL of each bacterial culture, incubated for 24 h at 37°C, and the presence of lysis zones or plaques was examined (Goodridge et al., 2003).

**Table 2:** **Strains tested against stock phages**.

**Source**	**Strains (amount)**	**Strains characteristics/description**
Food	*Escherichia coli* (10)	8 non-O157 STEC and 2 O157:H7 STEC
Stool sample	*Escherichia coli* (9)	4 O157:H7 STEC and 5 EPEC
	*Escherichia coli* (18)	Non-pathogenic
	*Shigella* spp.	Other enterobacteria
	*Salmonella* spp.	
	*Proteus mirabilis*	
	*Citrobacter freundii*	
	*Klebsiella pneumoniae* (17)	
Urine culture	*Escherichia coli* (17)	UPEC
ATCC	*Escherichia coli* (5)	35401; 43889; 43890; 43895 and 98222

*EPEC, enteropathogenic E. coli; O157:H7 STEC, O157:H7 Shigatoxigenic E. coli; non-O157 STEC, Shigatoxigenic non-O157 E. coli; UPEC, urophatogenic E. coli; ATCC, american type culture collection*.

### Meat assays

Beef from cow hindquarter purchased from retail was aseptically cut into pieces (1 cm^2^ of surface and 0.4 cm thick), placed in petri dishes and pre-equilibrated to 5 or 24°C. The required pH was obtained by washing with sodium chloride-magnesium sulfate (SM) buffer (0.05 M TRIS, 0.1 M NaCl, 0.008 M MgSO_4_, 0.01% w/v gelatin, pH = 7.5) prior to inoculation with bacteria and phage. Host strains employed in this study, namely non-O157 STEC (ARG4827; serogroup O18; harboring *stx*1 and *stx*2 genes) (Balagué et al., 2006), O157:H7 STEC (464; harboring *stx*1 and *eaeA* genes) and an EPEC (EPEC920; which harbors *eaeA* gene), were grown in Hershey medium supplemented with MgSO_4_ (5 mM) for 12 h at 37°C. Bacterial strains and specific bacteriophages added to the meat samples are detailed in Table 3. Twenty μL of each diluted bacterial suspension (ranging from 5.9 × 10^5^ to 3.9 × 10^7^ CFU/mL) were pipetted onto the surface of each meat piece and allowed to attach for 10 min at room temperature. Another 20 μL of each bacteriophage were then pipetted on the meat, at low multiplicity of infection (MOI), 1.7 × 10^9^ PFU/mL for DT1 and 1.4 × 10^9^ PFU/mL for DT6, or high MOI, 1.7 × 10^10^ PFU/mL for DT1 and 1.4 × 10^10^ PFU/mL for DT6. Pieces of meat were also added with SM buffer (pH 7.5), instead of phage suspension, as controls. At 3, 6, and 24 h, meat pieces were transferred to a sterile bag, 5 mL SM buffer were added and samples processed for 2 min in a Stomacher (Seward, London, UK). A 1 mL portion of the stomacher fluid was transferred to a sterile tube and cells were pelleted by centrifugation at 3000× *g* for 10 min. The supernatant was removed and cells were resuspended in 1 mL SM buffer. Finally, a 0.1 mL sample was removed, serially diluted (10^2^–10^4^-fold) in SM buffer and 0.1 mL volumes of each dilution were plated on Hershey agar for viable cell enumeration (Bigwood et al., 2008). Phage cocktail (DT1 and DT6 in equal proportions) was assayed on *E. coli* DH5α (indicator strain used for phage isolation) and in O157:H7 STEC (464) using the methodology employed for each individual phage described above. Three replicates were performed for each treatment and one meat piece processed for replicate. Uninoculated controls were tested to determine the presence of naturally occurring bacteriophages. Plaques (PFU/mL) were evaluated by the double layer agar technique (Jamalludeen et al., 2007).

**Table 3:** ***E. coli* viable cell logarithmic reductions after phage treatment of contaminated meat products**.

**Phage stock/sensitive strain**	**Assay conditions**		**Log reduction in *E. coli* viable cells^a^ after the incubation time (h)^b^**		
	**T (°C)**	**MOI**	**3**	**6**	**24**
DT1/EPEC (920)	5	4.4 × 10^2^	NS	^**^0.80 ± 0.14	NS
	24	4.8 × 10^2^	^**^0.30 ± 0.05	NS	NS
	5	4.4 × 10^1^	NS	^**^0.49 ± 0.09	NS
	24	4.8 × 10^1^	NS	NS	^**^0.46 ± 0.08
DT6/EPEC (920)	5	5.2 × 10^2^	^**^0.67 ± 0.12	^**^0.59 ± 0.11	^*^0.46 ± 0.15
	24	6.5 × 10^3^	^*^0.32 ± 0.09	NS	NS
	5	5.2 × 10^1^	NS	^*^0.30 ± 0.08	NS
	24	6.5 × 10^2^	NS	NS	NS
DT6/non-O157 STEC (ARG4827)	5	2.4 × 10^4^	^*^0.33 ± 0.09	^*^0.47 ± 0.12	^*^0.56 ± 0.17
	24	4.0 × 10^2^	^*^0.43 ± 0.13	NS	NS
	5	2.4 × 10^3^	NS	^*^0.37 ± 0.09	^*^0.50 ± 0.16
	24	4.0 × 10^1^	^*^0.35 ± 0.11	NS	NS
DT6/O157:H7 STEC (464)	5	2.3 × 10^3^	^*^0.59 ± 0.16	^**^0.86 ± 0.15	^*^0.38 ± 0.10
	24	5.8 × 10^3^	^**^0.77 ± 0.14	^***^1.15 ± 0.12	NS
	5	2.3 × 10^2^	^*^0.38 ± 0.09	^*^0.62 ± 0.18	NS
	24	5.8 × 10^2^	NS	^**^0.74 ± 0.13	NS
Cocktail/DH5α	5	2.25 × 10^4^	^*^0.91 ± 0.19	^**^2.16 ± 0.20	^**^2.23 ± 0.21
	24	1.75 × 10^4^	^*^0.66 ± 0.15	NS	NS
Cocktail/O157:H7 STEC (464)	5	1.56 × 10^5^	NS	NS	NS
	24	3.33 × 10^5^	^**^1.43 ± 0.24	^**^2.58 ± 0.21	^**^2.20 ± 0.22

MOI, multiplicity of infection (PFU/CFU); NS, not significant. Mean values of treated and control samples not significantly different using the scheffé method (

* significant at p = 0.05;

** significant at p = 0.01;

*** *significant at p = 0.001)*.

a *Log reduction in E. coli viable cells with respect to phage-free control*.

b *Mean of three data points ± standard deviations*.

### Bacteriophage insensitive mutants (BIMs) isolation

Bacteriophage insensitive mutants (BIMs) were isolated by the secondary culture method described by Guglielmotti et al. (2007) with some modifications. *E. coli* sensitive strains (one EPEC, three O157:H7 STEC and one non-O157 STEC) (A_600_ = 0.2−0.3) were infected with a phage suspension at different infection ratios (multiplicity of infection, MOI of ≈ 10 and 1), incubated in Hershey broth at 37°C for 24 h and observed visually until complete lysis. An uninfected culture of each *E. coli* strain was used as a control. Cultures exhibiting complete and delayed lysis were the best candidates to isolate BIMs. After lysis, further incubation for 48 h at 37°C was required for secondary growth. Each tube with secondary growth was spread on Hershey agar plates for colony isolation.

BIMs were isolated from meat as described in meat assays methodology described above modified with an extended incubation time (48 h) at 37°C. For both of the aforementioned methodologies, after incubation of agar plates, eight different colonies were randomly isolated (on agar plates) and cultured overnight in Hershey broth at 37°C. These isolates were purified by three consecutive streakings on Hershey agar plates. The growing colonies were isolated as presumptive BIMs.

### BIMs confirmation

Presumptive BIMs were confirmed by a liquid culture sensitivity test (Guglielmotti et al., 2007). Briefly, a log-phase culture (A_600_ = 0.2−0.3) of each presumptive BIM in Hershey broth was infected with the phage suspension at various MOI (≈ 10 and 1). Uninfected cultures of each *E. coli* strain were used as controls. BIMs cultures were incubated in Hershey broth at 37°C until growth of control strains was evident. Infected cultures that did not lyse at the first attempt were subcultured again. Each second subculture was prepared by transferring 2–3% of the final volume from the first culture to another test tube with 1 mL of fresh broth. When no bacterial lysis was evident, the resulting culture was stored at 4°C and subcultured under the same conditions. Presumptive BIMs that survived the third subculture were considered to be confirmed BIMs. Sensitivity of each parent strain (sensitive) was always determined in parallel to ensure lytic activity of phage suspensions.

### Determination of bacteriophage-insensitive mutant frequency, reversion, and stability

The emergence frequency of BIMs was evaluated by mixing the appropriate volume of an overnight culture of each strain (EPEC920 and O157:H7 STEC 464) and phage suspension (DT1 and DT6) to obtain a MOI of 100. The bacterium–phage mixture was supplemented with MgSO_4_ (5 mM), plated by the double-layer agar technique and incubated overnight at 37°C. BIM frequency was estimated as the ratio of the number of confirmed BIM to the initial bacterial number. All the experiments were performed in duplicate. Selected BIMs were propagated through 50 generations at 37°C and then checked by a plaque assay to evaluate reversion to phage sensitivity (O'Flynn et al., 2004).

Phage resistance stability was assayed by seven sequential subcultures of 2% portions of BIM cultures (Hershey broth) with independent addition of phage at each subculture (Guglielmotti et al., 2007). The loss of phage resistance was determined by comparing lysis of BIM culture with the control (mutant subculture without phage addition). The subculture where lysis first occurred was recorded.

### Statistical analysis

Means and standard deviations for data sets were calculated. Differences between means for control (untreated) and treated samples were compared by the Scheffé method and Origin 6.0 for graphics. Differences were considered statistically significant when *p*-values were <0.05.

## Results

### Isolation, viability, and characteristics of bacteriophage stocks

**Figure 1:**
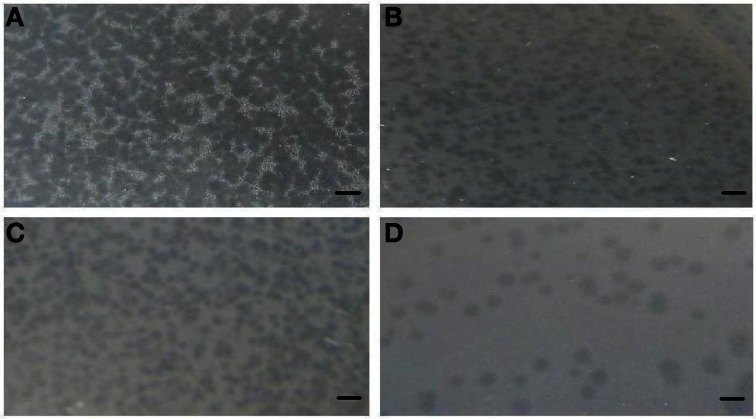
**Plaques produced by DT1 and DT6 on EPEC920 (A and B, respectively) and O157:H7 STEC strains (C and D, respectively).** Bars represent 1 cm.

**Figure 2:**
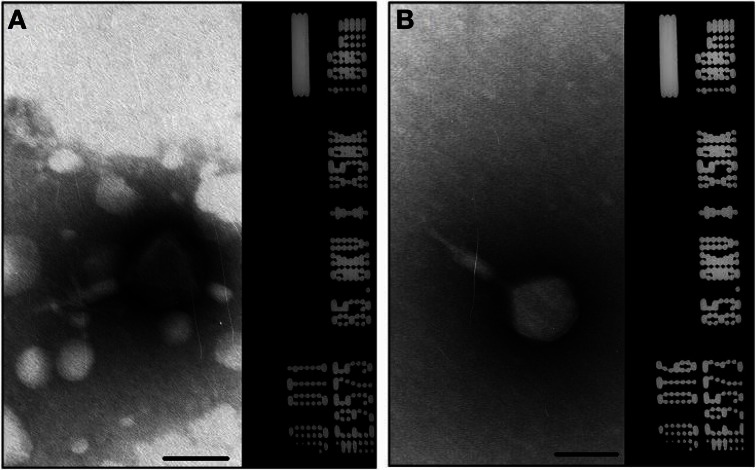
**Electron micrograph of phages DT1 (A) and DT6 (B) negatively stained with 2% phosphotungstic acid.** Dimensions are given in the text. Bars represent 100 nm.

A total of 10 bacteriophage stocks were obtained from diarrheic stool samples, titred and stored at 4°C. Figure 1 shows plaques produced by DT1 and DT6 on the EPEC920 and O157:H7 STEC strains. Phage concentration ranged between 1.2 × 10^10^ PFU/mL and 4.8 × 10^10^ PFU/mL. The viability of each bacteriophage stock stored at 4°C was evaluated after two months and similar titers were obtained, suggesting that the storage method used was adequate. Electronic microscopy allowed us to infer that bacteriophages DT1 and DT6, could taxonomically belong to T-even type of the *Myoviridae* family. Phages DT1 and DT6 had icosahedral heads and contractile tails. DT1 dimensions were of 89.3 nm (head diameter), 127.8 nm (tail length), 20.8 nm (tail thickness), and a total length of 217.1 nm; for DT6 measures were of 82.1 nm (head diameter), 125.7 nm (tail length), 17.7 nm (tail thickness), and 207.8 nm (total length) (Figure 2). PCR determinations of virulence factors (Stx1; Stx2; ST1; LT1 and Intimin) were negative for all bacteriophage stocks.

### Bacteriophage specificity

Non-*E. coli* and non-pathogenic *E. coli* strains were resistant to the lytic action of phages. Among the pathogenic *E. coli*, six (6) *E. coli* O157:H7 STEC, three (3) non-O157 STEC, five (5) EPEC, and two (2) UPEC strains were sensitive to phages evaluated (Table 4).

**Table 4:** **Host range of stock phages**.

**Phage stock**	***E. coli* sensitive strains**	**Total sensitive strains**
DT1	2 EPEC; 1 O157:H7 STEC;	4
	1 non-O157 STEC	
DT2	2 EPEC; 3 O157:H7 STEC	5
DT3	4 EPEC; 6 O157:H7 STEC;	13
	3 non-O157 STEC	
DT4	4 EPEC; 5 O157:H7 STEC;	12
	3 non-O157 STEC	
DT5	2 EPEC; 3 O157:H7 STEC	5
DT6	4 EPEC; 6 O157:H7 STEC;	13
	3 non-O157 STEC	
LM1	2 EPEC; 4 O157:H7 STEC;	7
	1 non-O157 STEC	
LM2	1 EPEC; 1 O157:H7 STEC;	4
	2 non-O157 STEC	
LM3	2 EPEC; 3 O157:H7 STEC;	8
	2 non-O157 STEC; 1 UPEC	
LM4	1 EPEC; 2 O157:H7 STEC;	6
	2 non-O157 STEC; 1 UPEC	

*EPEC, enteropathogenic E. coli; O157:H7 STEC, O157:H7 Shigatoxigenic E. coli; non-O157 STEC, Shigatoxigenic non-O157 E. coli; UPEC, uropathogenic E. coli*.

### Biocontrol tests on meat products

Two bacteriophages, which formed clearly defined plaques, with different host range were selected. The phage with the narrower range (DT1) was tested on an EPEC strain while the phage with the broader range (DT6) was tested on the same EPEC strain assayed previously and two STEC strains, one O157:H7 and one non-O157.

Significant decreases (*p* = 0.05) in viable cells (VC) for all tested strains were observed in comparison to the phage-free controls. Table 3 shows log VC significant reductions (*p* < 0.05) at high MOI in EPEC920, non-O157 STEC and O157:H7 STEC strain/DT6 systems after 3-h incubation, and in all phage/strain systems after 6-h incubation at 5°C. At high MOI values, VC reduction were still significant (*p* = 0.05) after 24 h of DT6 phage exposure. When the test was carried out at 24°C, significant VC reduction (*p* < 0.05) were observed after 3 h for DT1/EPEC920, DT6/O157:H7 STEC, DT6/EPEC920, and DT6/non-O157 STEC. These reductions were not maintained after 6 or 24 h, with the exception of the 1.15 log reduction obtained for DT6/O157:H7 STEC after 6 h.

When the results obtained with different MOIs were compared, significant differences (*p* = 0.05) at both temperatures were observed for most phages/bacterium combinations. Reductions in a phage/bacterium system were higher at the highest temperature, up to 0.29 log (1.15 log – 0.86 log) CFU for DT6/O157:H7 STEC at 6 h, with exception in a single case for the DT6/EPEC920 system, after 3-h incubation.

**Figure 3:**
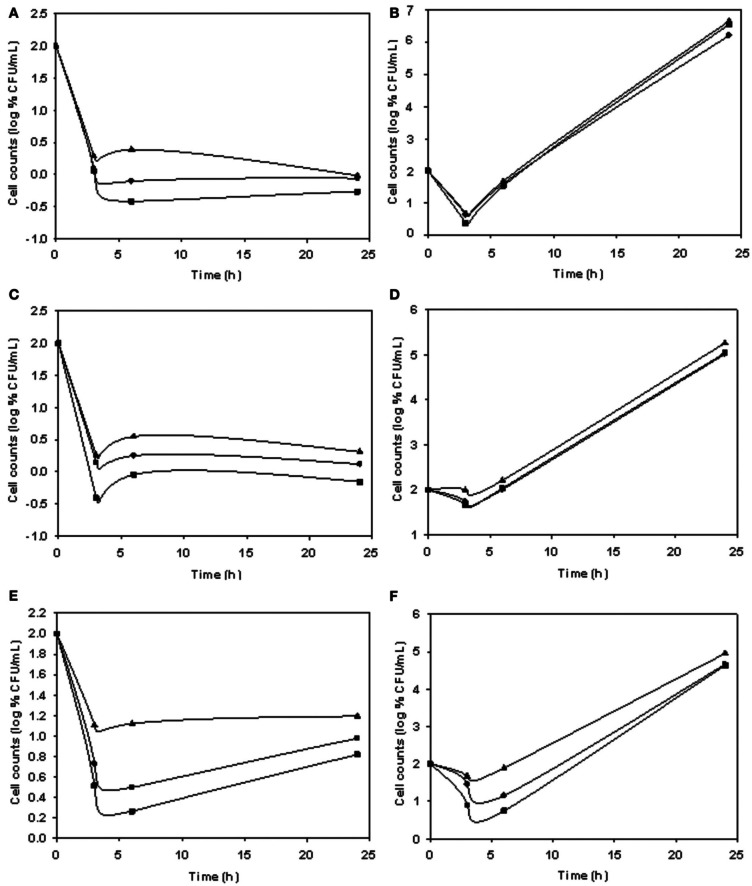
**Comparison of the lytic ability of phages at different MOI and temperatures.** Symbols represent phage free controls (▲), low MOI (•), and high MOI (■). Cell count (CFU/mL) using as matrix inoculated meat with DT1/EPEC920 at 5°C **(A)** and 24°C **(B)**, DT6/EPEC920 at 5°C **(C)** and 24°C **(D)** and DT6/O157:H7 STEC at 5°C **(E)** and 24°C **(F)**.

Phage stocks displayed different host range (Table 4), some having limited and others having a broader range. The lytic ability of phages at different MOI values and temperatures is shown in Figure 3. The biocontrol effectiveness against EPEC920 shown by DT1 (Figures 3A,B) and DT6 (Figures 3C,D) phages was different only at the lower temperature. At 5°C, DT1 produced a greater reduction in the number of VC after 6-h incubation than DT6, but DT6 biocontrol over EPEC920 was faster and prolonged. At 24°C there was no difference in the lytic effects of phages with the exception at the longer incubation time and lower MOI (4.8 × 10^1^ PFU/CFU) where DT1 was more effective. DT6 was also active against O157:H7 strains; being more effective at 24°C (Figure 3F) though with a prolonged lytic action at 5°C (Figure 3E).

**Figure 4:**
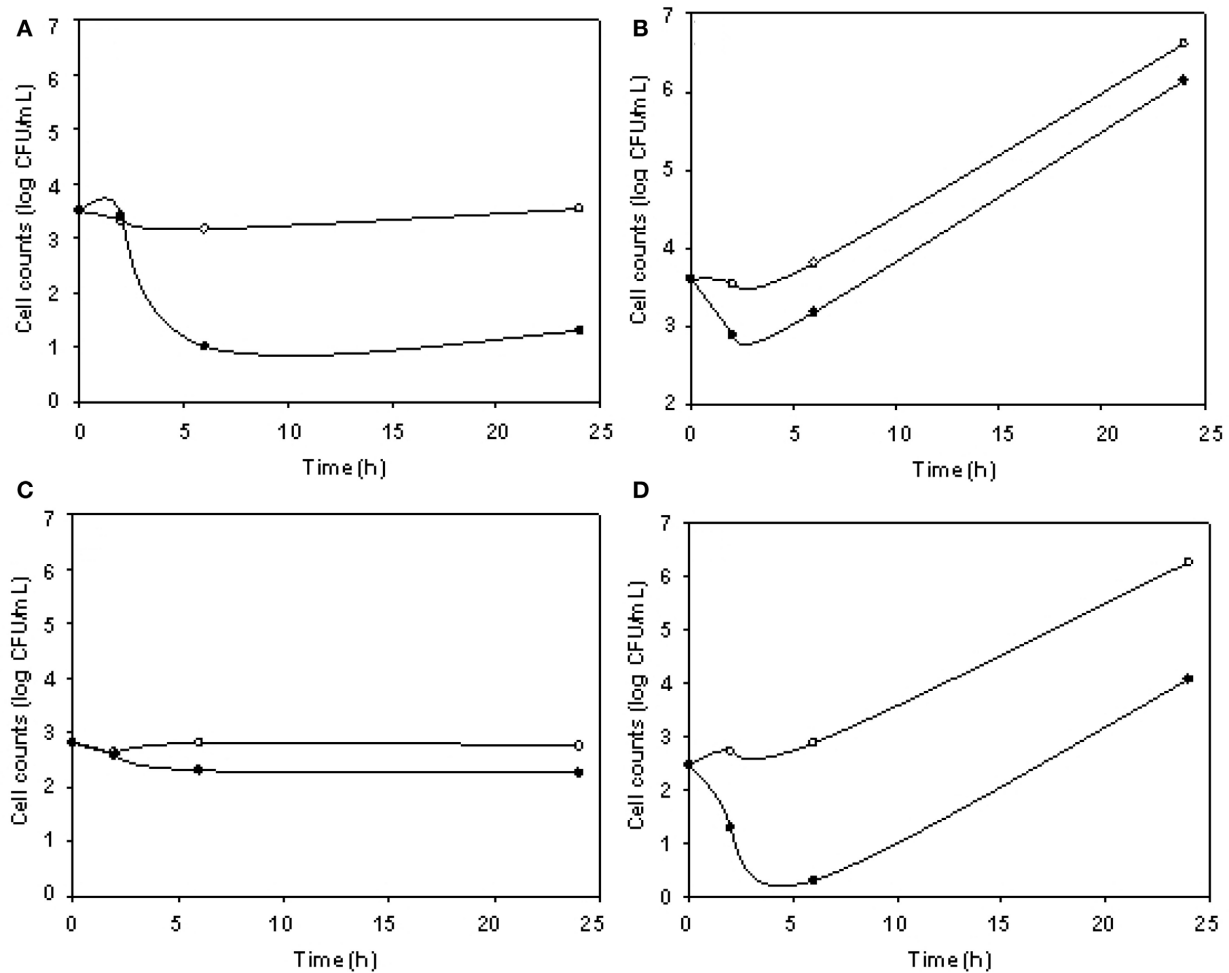
***E. coli* viable cell counts (CFU/mL) in absence (°) and presence (•) of phages (cocktail) in meat products.** Cocktail/DH5α at 5°C **(A)**, cocktail/DH5α at 24°C **(B)**, cocktail/O157:H7 STEC at 5°C **(C)**, and cocktail/O157:H7 STEC at 24°C **(D)** systems.

The phage cocktail successfully reduced DH5α VC only at 5°C, while for O157:H7 STEC reductions took place only at 24°C (Figure 4). DH5α was significantly reduced at 3, 6, and 24 h, being 2.23 log the major reduction value obtained at 24 h. For O157:H7 STEC, VC reductions up to 2.58 log at 6 h were observed, in addition, phage cocktail was able to achieve an effective and prolonged biocontrol effect (2.20 log at 24 h) (Table 3).

### Isolation and reversion of BIMs

After incubating meat inoculated with phage/bacteria systems at 24°C for 24 h, significant bacterial growth was observed. To assess whether bacterial re-growth on meat experiments may be due to resistance, eight (8) potential BIMs were isolated from meat incubated for 48 h at 37°C and only 2 (25%) were confirmed as BIMs by the liquid culture sensitivity test method. Another eight (8) presumptive BIMs were isolated by the secondary culture method and 6 (75%) were confirmed. BIM frequencies observed for all the evaluated strains were low, ranging from 6.5 × 10^−7^ to 1.8 × 10^−6^ (Table 5).

**Table 5:** ***In vitro* and meat BIM isolation and determination of BIM frequency**.

**Source of BIMs**	**Presumptive BIMs tested**	**Confirmed BIMs (%)**	**BIM frequency (mean ± SD)^a^**
Secondary culture	8 EPEC	6 (75.0)	EPEC/DT1 = 8.7 × 10^−7^± 3.9 × 10^−8^
			EPEC/DT6 = 6.5 × 10^−7^ ± 4.8 × 10^−8^
Meat	8 O157:H7	2 (25.0)	O157:H7 STEC/DT1 = 1.8 × 10^−6^ ± 1.5 × 10^−7^
	STEC		O157:H7 STEC/DT6 = 1.3 × 10^−6^ ± 9.4 × 10^−7^

a *SD, standard deviation. Means and SD were calculated from duplicate experiments*.

*EPEC, enteropathogenic E. coli; O157:H7 STEC: O157:H7 Shigatoxigenic E. coli*.

One BIM, which could form a lawn, was found to be susceptible to phage DT6. BIMs evaluated for phage DT1 were resistant and showed no reversion to phage sensitivity. Mutants isolated from EPEC920 maintained their phenotype up to the fifth subculture. Mutants isolated from O157:H7 STEC maintained resistance up to the fourth subculture and only one was resistant at the seventh subculture.

## Discussion

Biocontrol by bacteriophages in meat products was developed in the last years and their recent “generally recognized as safe” (GRAS) designation and approval as food additives (FDA, 2006) has opened the discussion about “edible virus.” Phages are naturally present in significant numbers in water and food (Sulakvelidze, 2011). In fresh meats and meat products more than 10^8^ viable bacteriophages per gram may be present (Kennedy and Bitton, 1987), implying that phages are commonly consumed in large numbers. In addition, bacteriophages are especially abundant in the gastrointestinal tract (Breitbart et al., 2003; Hitch et al., 2004). However, most phage-host systems are highly specific, which is a general limitation (Carlton et al., 2005). Phage must be lytic and non-transducing as minimum requirements to ensure safety (Greer, 2005). Nevertheless, application of bacteriophages is a non-destructive, natural, self-perpetuating biocontrol method and can be as efficient as chemical agents for controlling specific bacterial pathogens (Leverentz et al., 2001). On the other hand, the high specificity of phages allows direct application onto a product or the possibility to use bacteriophages as “food additives,” mixing them with foods without affecting their quality (Jay, 1996), hygiene and other normal microbiota viability of the food (Kudva et al., 1999) or the consumers' (Chibani-Chennoufi et al., 2004).

Previous studies have demonstrated the feasibility of isolating phages that specifically lyse O157:H7 *E. coli* strains (Sulakvelidze and Barrow, 2005; Raya et al., 2006). O'Flynn et al. (2004) developed a three-phage cocktail that was effective for reducing the numbers of *E. coli* O157:H7 on meat products. On the other hand, in a similar experiment, Dykes and Moorehead (2002) found no effect of bacteriophage for control of *Listeria monocytogenes* development on contaminated beef, however, this treatment did not yield an appreciable reduction due to the low MOI used. Also, phage-mediated reductions of bacterial cell viability have been reported in various food matrices like contaminated melon (Leverentz et al., 2003) and cheese (Carlton et al., 2005; Bueno et al., 2012).

Phages isolated in this study were characterized by their host range, electron microscopy (DT1 and DT6), and PCR analysis. The host range evaluation demonstrated that all O157:H7 strains, most non-O157 STEC and some EPEC isolated from diarrheic faeces or food, were sensitive to one or several bacteriophages. In contrast, no nonpathogenic strain was affected. These finding suggest the possibility of using phages for food conservation without altering the gastrointestinal tract normal microbiota. However, a broader host range needs to be assessed to ensure safety for commensal bacteria. Phages did not contain genes encoding *stx1*, *stx2*, *eaeA*, *LT1* and *ST1*, but other virulence factors and phage-encoded genes may contribute to bacterial virulence, so further sequencing and bioinformatic analysis are required to ensure they are benign prior to their use as biocontrol tools.

DT1 and DT6 on meat, gave statistically significant VC reductions, comparable to those previously reported (O'Flynn et al., 2004). EPEC920 was tested with both phages individually; at 5°C DT6 produces a minor reduction in VC count but a rapid and prolonged effect in time. This observation may be related to different adsorption rate and lysis time distinctively influenced by environmental conditions (Shao and Wang, 2008). Higher reductions were observed (for O157:H7 STEC) at the higher temperature (24°C), probably due to the active growth of bacteria allowing an efficient bacteriophage replication. Similar results at 5 and 24°C for *Salmonella* and *Campylobacter* were described by Bigwood et al. (2008). The biocontrol effect for all phages analyzed was found to be dose-dependent, with the highest phage concentration being the most effective, as was also found by Leverentz et al. (2001). Our findings showed that phage-bacteria interaction on meat surfaces was significantly influenced by the initial number of inoculated phage and bacteria. Atterbury et al. (2003) obtained marginal reductions of approximately 1 log CFU only with high initial bacterial density of *C. jejuni* (4–6 log CFU). The requirements for a relatively high threshold density of bacterial host cells may limit the impact of phages on bacteria, constituting an important impediment to phage biocontrol (Greer, 2005). In addition, there are several factors influencing inactivation on food, the most relevant in our experiments is the food matrix ability to absorb liquid from the phage suspension. This is a decisive parameter which physically limits the distribution of phage particles in order to reach all targeted bacteria. Therefore, decreased effectiveness of these treatments may be partially due to limited diffusion and contact between bacteria and phage particles. Moreover, targeted bacteria may be embedded within the rather complex food matrix, thereby shielding them from phage particles. On these grounds, a greater biocontrol effect may be achieved by modifying phage application, e.g., by using larger liquid volumes to avoid total liquid absortion. While the results were statistically significant we cannot ascertain the practical use of these phages individually on meat products, mainly due to the 1.15 log reduction obtained at best. However, the phage cocktail (containing DT1 and DT6) at a higher MOI was able of further reduce viable cell counts of O157:H7 STEC (464) than individual phages. This indicates that phages may be of practical value if cocktails contain higher numbers of different phages (to reduce BIMs emergence and expand cocktail host range) and higher MOI values, since others authors report that working with higher phage concentrations generally resulted in greater inactivation (Guenther et al., 2009). In addition, DH5α indicator strain was only significantly reduced at 5°C, this reduction may be caused by lysis from without since DH5α has a shorter lipopolysaccharide (LPS), thereby being more permissive and susceptible to this mechanism, while for O157:H7 STEC (464) at 5°C this mechanism was not observed and no reduction was obtained. This may rather be due to the absence of bacterial growth necessary for phage replication. At 24°C, DH5α cell number was not reduced (only after a 3 h of incubation a 0.66 log reduction was observed) and re-growth was observed. This recovery in cell number may be due to cells escaping phage treatment by limited phage diffusion and a subsequent multiplication in addition to BIMs emergence.

In our trials we found an increase in VC number after 24 h at 24°C, suggesting that there is a potential phage-resistant variant selection under these testing conditions. Thus, we propose to analyze the existence of *spontaneous bacteriophage*-*insensitive mutants (BIMs)*, naturally present in bacteria, in order to evaluate if they could prevent the use of phages to improve food safety. Other authors have also reported a subsequent bacterial growth during the *in vitro* challenge test (O'Flynn et al., 2004). BIMs emergence, which could compromise the efficacy of a phage treatment, is often associated with point mutations in genes encoding receptor molecules on the bacterial cell surface and commonly revert to phage sensitivity rapidly (Garcia et al., 2007). Kudva et al. (1999) propose that low temperatures and absence of bacterial growth favor phage adsorption and infection. In contrast, higher temperatures, cell growth, and the potential for phenotypic variability in expression of the O-antigen favor survival of phage-resistant cells. We were able to isolate *E. coli* BIMs from meat assays and, at a higher rate, when using the secondary culture method. BIMs isolated in this study were tested to evaluate resistance phenotype stability and reversion. As previously reported for other systems (O'Flynn et al., 2004), all these BIMs present low emergence frequency, variable stability, and reversion, namely, mutants resistant to phage DT6 revert while those resistant to DT1 show no reversion to phage sensitivity. These findings suggest that *E. coli* BIMs should not prevent the use of phages as biocontrol tools, mainly due to the low emergence frequency observed for all phages evaluated. In our trials phage cocktail reduced the appearance of presumptive-BIMs, however, further studies are required in order to evaluate the optimal conditions allowing the reduction of BIM emergence such as adding more different phages in the cocktail mixture.

We have isolated 10 bacteriophages belonging to the T-even type of the *Myoviridae* family. Phages isolated in this study were negative for all the virulence factors assayed and presented lytic activity for different *E. coli* virotypes. In addition, all nonpathogenic strains evaluated were not affected. BIMs isolated by exposure to DT1 and DT6 showed low emergence frequency and in *in vitro* challenge tests VC reductions were highly significant (up to 6.3 log units) (data not shown). However, on pieces of beef assayed, reduction obtained with individual phages was low and the phage cocktail showed greater reductions although lower than expected.

### Conflict of interest statement

The authors declare that the research was conducted in the absence of any commercial or financial relationships that could be construed as a potential conflict of interest.
